# Metagenomic analysis of *Aedes aegypti* and *Culex quinquefasciatus* mosquitoes from Grenada, West Indies

**DOI:** 10.1371/journal.pone.0231047

**Published:** 2020-04-13

**Authors:** Maria E. Ramos-Nino, Daniel M. Fitzpatrick, Korin M. Eckstrom, Scott Tighe, Lindsey M. Hattaway, Andy N. Hsueh, Diana M. Stone, Julie A. Dragon, Sonia Cheetham

**Affiliations:** 1 Department of Microbiology, Immunology, and Pharmacology, School of Medicine, St. George’s University, Grenada, West Indies; 2 Department of Pathobiology, School of Veterinary Medicine, St. George’s University, Grenada, West Indies; 3 University of Vermont Massively Parallel Sequencing Facility, Burlington, Vermont, United States of America; University of Maryland, UNITED STATES

## Abstract

The mosquitoes *Aedes aegypti* (Linnaeus, 1762) (Diptera: Culicidae) and *Culex quinquefasciatus* Say, 1823 (Diptera: Culicidae) are two major vectors of arthropod-borne pathogens in Grenada, West Indies. As conventional vector control methods present many challenges, alternatives are urgently needed. Manipulation of mosquito microbiota is emerging as a field for the development of vector control strategies. Critical to this vector control approach is knowledge of the microbiota of these mosquitoes and finding candidate microorganisms that are common to the vectors with properties that could be used in microbiota modification studies. Results showed that bacteria genera including *Asaia*, *Escherichia*, *Pantoea*, *Pseudomonas*, and *Serratia* are common to both major arboviral vectors in Grenada and have previously been shown to be good candidates for transgenetic studies. Also, for the first time, the presence of Grenada mosquito rhabdovirus 1 is reported in *C*. *quinquefasciatus*.

## Introduction

The mosquito species *Aedes aegypti*. and *Culex quinquefasciatus* are a public health concern due to their ability to be vectors of many arboviruses. *Aedes aegypti*, for example, transmits chikungunya virus, Zika virus, yellow fever virus, and dengue virus, one of the most rapidly spreading vector-borne pathogens in the world with 2.5 billion people at risk of infection and approximately 500,000 people developing severe dengue disease annually [1]. *Culex quinquefasciatus*, on the other hand, is capable of transmitting arboviruses like West Nile virus (WNV), the leading cause of mosquito-borne disease in the continental United States [2], as well as nematodes affecting human and animal health (lymphatic filariasis, dirofilariasis etc.) [3–5]. Currently, personal protective measures and control of mosquito populations are the only available strategies to prevent arboviral diseases because there are no therapeutic treatments for arboviruses and vaccines are limited. Organizations are constantly facing challenges with the use of conventional vector control methods because of sustainability, organizational complexity [6], and the rise of insecticide resistance [7–9].

Manipulation of mosquito microbiota is emerging as a promising method for the development of vector control strategies. Some of these strategies include: 1) The introduction of microorganisms that interfere with the pathogens within the vector. Examples of this strategy include the use of entomopathogenic fungi such as *Beauveria bassiana* and *Beauveria brongniartii* [10], and the alpha-proteobacteria *Wolbachia* [11–13]. *Wolbachia* is a natural intracellular bacterial symbiont maternally transmitted to offspring that can induce cytoplasmic incompatibility, where mating between *Wolbachia*-infected males and uninfected females yields eggs that fail to develop. Also certain *Wolbachia* strains may cause a decrease in the vectorial capacity by interfering with vector competence or by shortening vector lifespan [14]. 2) Another vector control strategy is the use of symbiotic bacteria that are transformed to express effector molecules for use in paratransgenic approaches. For example, *Metarhizium anisopliae* has been genetically transformed to express the anti-*Plasmodium* effector molecules SM1 and scorpine, which are both reported to interfere with *Plasmodium falciparum* in *Anopheles* mosquitoes [15].

*Aedes aegypti* and *C*. *quinquefasciatus* are the two predominant anthroponotic mosquitoes in Grenada, West Indies, [16]. *Aedes aegypti* transmits dengue, Zika, and chikungunya locally. No major human pathogens transmitted by *C*. *quinquefasciatus* have been reported in Grenada, but the mosquito is capable of transmitting several arboviruses that occur in other Caribbean islands (e.g., WNV, St. Louis Encephalitis virus) or in neighboring mainland South American countries (e.g., *Wuchereria bancrofti*) [17]. The microbial composition of *A*. *aegypti* and *C*. *quinquefasciatus* mosquitoes has been previously studied [18,19,20–27,28–36]. However, several arthropod-borne diseases that are endemic to the mainland Americas are not believed to be established in Caribbean islands (e.g. Mayaro virus, Oropouche virus, *Wuchereria*) [17,37–40]. While variation in climate and the makeup and behavior of local mosquito species may explain this in part, regional differences in local mosquito microbiota may also contribute to differences in their competence as vectors of pathogens of human and animal interest [6,33,41]. Metagenomic analyses have recently been used to identify novel arboviruses in Caribbean mosquitoes [19,42]; little else is known about these viruses. It is thus important to characterize the microbiome of mosquitoes on a regional level to identify heretofore unknown organisms, including agents that can be studied further for their effects on vector competence and their use in vector control studies. Through the use of metagenomics, this study will shed some light on: 1) the identification and determination of the relative estimated abundance (REA) of microbiota for these two arboviral vectors; 2) the identification of microbiota similarities and unique microbiota elements of these two mosquitos to provide potential targets for developing mosquito/arbovirus control strategies; and 3) the identification of microorganisms not yet known to occur in mosquitoes in Grenada.

## Material and methods

### Mosquito collection

**Fig 1:**
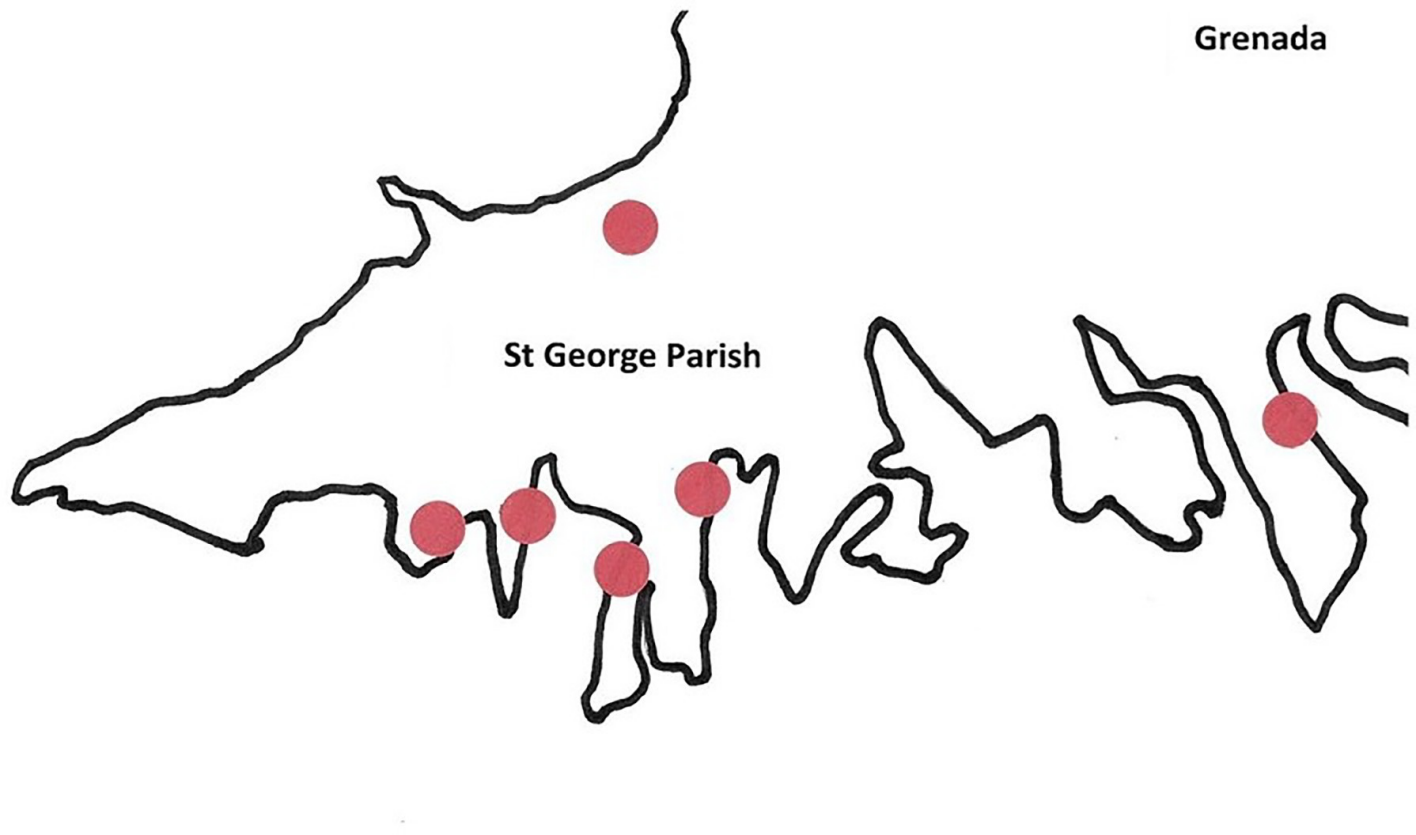
Map of collection sites in St. George parish-Grenada. Six sites were used during the year of collecting mosquitoes [47].

Three hundred *A*. *aegypti* mosquitoes and 300 *C*. *quinquefasciatus* were randomly selected out of 1,152 *A*. *aegypti* and 3,000 *C*. *quinquefasciatus* collected between January 2018 and December 2018 from six semi-rural locations in St. George Parish (Fig 1), the most populated parish in Grenada (12°15'46'' N 61°36'15'' W). The six collection sites were within an approximately five square-mile area and were chosen for their proximity to the capital city (which contains the main seaport), the airport, most major marinas, and for their high density of people. Traps (Biogents Sentinel, Biogents, Regensburg, Germany) were baited with octenol and yeast-based carbon dioxide attractants, as previously described [43]. In brief, at each of the six sites, one trap was deployed twice weekly within three meters of a house and was collected after 24 hours. Mosquitoes were dispatched at −80°C and identified to species by morphological analysis. Identification keys in Darsie and Ward (2013) [44] were used to discriminate between species known to occur in Grenada based on the Walter Reed Biosystematics Unit (2019) [45]. Mosquitoes were placed in RNAlater® (Sigma Aldrich, St. Louis, Missouri, USA) after identification for later processing. Mosquito heads were removed before RNA extraction to prevent PCR inhibition [46]; wings and legs were also removed to reduce host (mosquito) RNA. No specific permissions were required for this study since it was carried out in private lands. The study did not involve endangered or protected species. No IACUC was required for the use of mosquitoes in this study.

### Total RNA extraction and RNA-Seq

RNA extraction was performed in batches of 30 mosquitoes at a time (ten pools) using TRIzol (ThermoFisher, Carlsbad, California, USA). Invitrogen^™^ Phasemaker^™^ Tubes (ThermoFisher) were used for the phase separation. RNA was DNase-treated using TURBO DNA-*free*^™^ (ThermoFisher) and RNA quality evaluated utilizing an Agilent 2100 Bioanalyzer (Agilent, Santa Clara, California, USA) as previously described [48]. All sub-pools were pooled again for library construction, which was performed using the NuGEN Tecan universal RNA sequencing reagents as recommended by the manufacturer.

**Fig 2:**
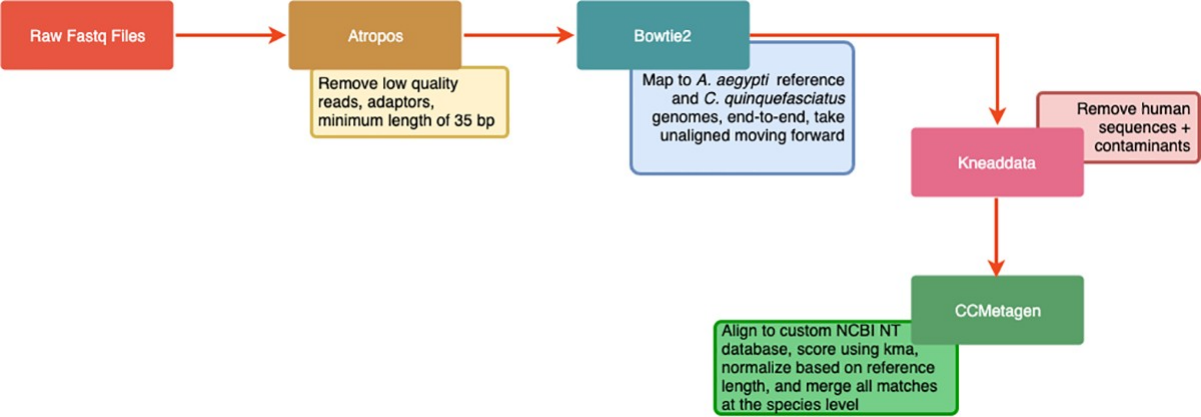
Metagenomic data analysis flow chart.

The metagenomic analysis flow used in this study can be found in Fig 2. Briefly, shotgun metagenomic sequencing was run using the Illumina HiSeq 1500 for deep sequencing. Raw fastq files were assessed for quality using Illumina FastQC version 0.11.8. Trimming and quality filtering of reads was performed using Atropos (https://omictools.com/atropos-tool), removing Illumina universal adaptors, reads with base calls below Q20, and reads with a length less than 35 bp. Additional host read removal was performed in silico using Bowtie2 (v. 2.3.4.3). Reads were mapped to their respective hosts, either the *A*. *aegypti* reference genome, assembly AaegL5, available at https://www.vectorbase.org/organisms/aedes-aegypti or the *Cx*. *quinquefasciatus* reference genome, assembly GCA_000209185.1 available at https://www.ebi.ac.uk/ena/data/view/GCA_000209185.1, using end-to-end read alignment. These non-mosquito reads were analyzed using CCmetagen (https://www.biorxiv.org/content/10.1101/641332v1) which uses the June 2019 version of the NCBI nt database excluding all taxids for environmental eukaryotes and prokaryotes, unclassified sequences, and artificial sequences in order to avoid misclassification based on contaminants. CCMetagen uses a weighted mapping approach based on the kma aligner in order to improve taxonomic classification of regions that are highly similar across microbial genomes. Abundance of the microorganisms, after metagenomic analysis, is expressed as the number of nucleotides mapped to the reference, normalized by the length of the reference in order to correct for differences in genome size. The relative estimated abundance (REA) reflects the percentage of the total abundance. Metagenomic data for *Aedes aegypti* and *Culex quinquefasciatus* is deposited in https://www.ncib.nlm.nih.gov/sra/PRJNA564787.

### RT-PCR verification

Approximately 200 ng of total RNA per pool (ten sub-pools) was reverse transcribed using a High-Capacity cDNA Reverse Transcription Kit (ThermoFisher). RT-PCR conducted on the cDNA was produced using previously published specific primers (S1 Table). PCR amplicons of expected size were extracted from gels using the QIAquick Gel Extraction Kit (QIAgen, Hilden, Germany) following the manufacturer’s protocol. Amplicons were sent to the Molecular Cloning Lab, San Francisco, California (https://www.mclab.com/) for direct Sanger sequencing. Raw sequence data were manually edited using Chromas 2.6.5 software and then compared with the sequence database using the NIH’s Basic Local Alignment Search Tool (BLAST). Sequences of microorganisms were aligned with Clustal Omega (https://www.ebi.ac.uk/Tools/msa/clustalo/) to obtain a consensus sequence. Primers used in this project are included in S1 Table [49–51].

### Isolation of microorganisms and taxonomic assignment

Freshly collected *A*. *aegypti* and *C*. *quinquefasciatus* were surface cleaned and then dissected to obtain the salivary glands and midguts. Mosquitoes were serially rinsed in sterile phosphate-buffered saline (PBS), followed by ethanol (70%), and finally rinsed three times in PBS. Aliquots of 100 μl from the last PBS washes were plated on blood agar plates as control groups of the surface cleaning process. Mosquitoes were dissected under a microscope, and salivary glands and midguts were collected in sterile PBS and macerated with a pestle. An aliquot of 100 μl was transferred to blood agar plates and incubated at 28°C for 24–48 h, followed by DNA isolation of individual colonies using the Qiagen DNeasy Blood and Tissue kit. Universal bacterial 16s rRNA primers (see S1 Table) were used to amplify a 465 bp product [49] by PCR. Amplicons of the expected size were purified using the QIAquick Gel Extraction Kit (Qiagen) and Sanger-sequenced. Editing and assignment of a bacterial taxonomic hierarchy was done as described above. Isolates were stored as glycerol stocks at -80°C.

## Results

The metagenomic analysis of both *A*. *aegypti* and *C*. *quinquefasciatus* in Grenada showed a microbiome composed primarily of bacteria (75.22% and 96.42% REA, respectively) (Table 1, S1A/*Aedes* Fig, S1B/*Culex* Fig).

**Table 1:** *Aedes aegypti* and *Culex quinquefasciatus* common microbiota in Grenada (Relative estimated abundance).

Organism					*Aedes*	*Culex*
**Bacteria**					***75*.*22***	***96*.*42***
** **	**Phylum**	**Class**	**Family**	**Genus**	** **	** **
** **	Proteo-bacteria				**81.28**	**94.92**
** **		Gamma-proteobacteria	*Enterobacteriaceae*	*Escherichia*	64.52	11.15
** **			* Enterobacteriaceae*	*Serratia*	8.28	0.01
** **			*Halomonadaceae*	*Zymobacter*	1.66	1.50
** **			*Pseudomonadaceae*	*Pseudomonas*	1.03	0.02
			*Xanthomonadaceae*	*Stenotrophomonas*	0.66	0.01
			*Moraxellaceae*	*Acinetobacter*	0.52	0.14
			*Halomonadaceae*	*Carnimonas*	0.48	0.45
			*Halomonadaceae*	*Halotalea*	0.12	0.09
			*Erwiniaceae*	*Pantoea*	0.09	0.05
			*Xanthomonadaceae*	*Xanthomonas*	0.07	0.01
** **		Alpha-proteobacteria	*Acetobacteriaceae*	*Asaia*	0.22	0.02
** **	Actino-bacteria			* *	**16.7**	**2.81**
** **		Actinobacteria	*Actinomycetaceae*	*Actinomyces*	16.7	2.81
** **	Spirochaetes		* *	* *	**1.54**	**1.94**
** **		Spirochaetia	*Spirochaetaceae*	*Spironema*	0.94	1.18
	Firmicutes				**0.28**	**0.26**
		Bacilli	*Leuconostocaceae*	*Leuconostoc*	0.1	0.02
**Fungi**					*0*.*19*	*0*.*10*
**Parasite**					*0*.*08*	*1*.*89*
**Virus**					*24*.*13*	*1*.*58*

The phylum Proteobacteria dominated the bacteria in both mosquitoes (81.28% REA in *A*. *aegypti* and 94.92% in *C*. *quinquefasciatus*) (Table 1, S2A1/*Aedes* Fig, S2B1/*Culex* Fig). Of the Proteobacteria, *Escherichia* (64.52% REA), a Gammaproteobacteria belonging to the *Enterobacteriaceae* family was predominant in *Aedes*. In *Culex*, the genera *Wolbachia* (62.79% REA), an Alphaproteobacteria belonging to the *Anaplasmataceae* family was the predominant bacteria, followed by *Escherichia* (11.15% REA) (S2A2–S2A4/*Aedes* Fig, S2B2–S2B4/*Culex* Fig).

Common bacteria found in both mosquitoes included the genera *Escherichia*, *Actinomyces*, *Zymobacter*, *Spironema*, *Carnimonas*, *Acinetobacter*, *Halotalea*, *Pantoea*, *Leuconoctoc*, *Pseudomonas*, *Asaia*, *Stenotrophomonas*, *Xanthomonas*, and *Serratia*. *Actinomyces* was the most abundant bacteria in both mosquitoes after *Escherichia* (Table 1). Unique to *Culex* were the genera *Wolbachia*, *Arcobacter*, *Aeromonas*, *Burkholderia*, *Holospora*, *Salmonella*, *Erwinia*, *Anaplasma*, and *Fructobacillus*. The bacteria genera *Pseudoxanthomonas* and *Halomonas* were unique to *Aedes* mosquitoes (S2A4/*Aedes* Fig,S2B4/*Culex* Fig).

The genus *Asaia* which was present in the metagenomic analysis of both *Aedes* and *Culex*, has unique features such as presence in organs of female and male mosquitoes and vertical and horizontal transmission. These characteristics lead to the possibility of introducing it as a robust candidate for vector control *via* paratransgenesis. Because it is vital to be able to culture bacteria for paratransgenic approaches, this bacterium was further tested for culturability and location in the vector [52]. *Asaia* was isolated by simple routine culture methods from both *Aedes* and *Culex*. Culture of the salivary glands and midguts of individual mosquitoes resulted in the isolation of *Asaia* from both the salivary glands and midguts of *Aedes*, and only from the guts of *Culex*.

A large number of reads were unclassified at the genera level for the fungi and the parasites. The fungi kingdom was only 0.19% and 0.1% REA for *Aedes* and *Culex* respectively. The family *Sclerotiniaceae* was common to both mosquitoes (7.75% REA in *Aedes* and 12.8% in *Culex*) and dominated in *Culex*, while the family *Aspergillaceae* (44.6%) was dominant in *Aedes* (S3A1–S3A2/*Aedes* Fig,S3B1 and S3B2/*Culex* Fig).

The parasites were also very rare with 0.08% in *Aedes*, and 1.86% REA in *Culex* with *Trypanosomatidae* as the dominant family in *Culex* and the *Albuginaceae* and *Lecudinidae* families dominant in *Aedes* (S4A1 and S4A2/*Aedes* Fig,S4B1 and S4B2/*Culex Fig*). Common to both mosquitoes was the genus *Albugo*.

Subsequent RT-PCR assays were performed on ten sub-pools of *C*. *quinquefasciatus* mosquitoes in order to confirm the presence of the trypanostomatids detected in the metagenomics analysis. All primers and references are listed in S1 Table. Amplicons of the correct size were produced in four of the ten sub-pool PCRs (S5 Fig). Three Sanger-sequenced amplicons had identical sequences to each other while the fourth failed to sequence. The best species-level match was *Paratrypanosoma confusum*, a recently characterized trypanostomatid that is likely insect-specific (identity: 99.7% to GenBank accession number KF963538.1) [53].

The dominant viruses in *Aedes* were described in previous publication [47]. Due to the limitations in the databases presently available, a large amount of reads for viruses from the metagenomic analysis remained un-classified, but for *Culex*, the *Circoviridae* (33.41% REA) family, particularly the genus *Circovirus* was the most dominant. The next most abundant family in Culex was *Rhabdoviridae*, dominated specifically by Grenada mosquito rhabdovirus 1. Finally, the third most abundant virus family for *Culex* was *Flaviviridae* dominated by Culex flavivirus (S6A1 and S6A2/*Aedes* Fig,S6B1 and S6B2/*Culex* Fig).

RT-PCR using hemi-nested pan-flavivirus primers produced amplicons of the expected size in nine of ten sub-pools (S1 Table, S7 Fig). Sanger sequencing of two of the amplicons confirmed infection with Culex flavivirus (identities: 98.8% and 99.2% to GenBank accession number MH719098.1), while the other seven amplicons produced overlapping reads, suggesting a mixed infection.

Two viruses with unknown families were also detected in *Culex*: Culex phasma-like virus and Terena virus.

## Discussion

The understanding of an organism can no longer be assessed in isolation, but rather needs to be viewed as a complex that includes its community of associated microorganisms and their interactions [27]. With this in mind, our study goals were to identify and compare the microbiota of two important vectors of mosquito-borne pathogens in Grenada, *Aedes aegypti* and *Culex quinquefasciatus*. Studies on the role of microbial communities in the mosquito biology and pathogen interference has led to the development of new vector control approaches based on microbiota modifications [54]. It has also been shown that environmental factors influence the microbial composition of breeding sites and food resources (plant materials, water sources, blood) [23,27,55] that become part of the adult mosquitoes’ microbiome. Similarly, the microbiome for a given mosquito species can differ based upon geography and parental lineage [6,33,41]. Thus, it is important that regional studies are conducted, as microbiome region-specific vector control approaches may be required. Our results could lead to future studies on the use of these organisms in mosquito control projects in Grenada.

Our results were similar to several other studies [6,33,41], despite differences in techniques used for identification and characterization of microbiota. For instance, microbiota in Grenadian mosquitoes is dominated by bacteria, particularly those associated with the gut, an observation confirmed in other studies [56]. In *Aedes aegypti* adult mosquito for example, Proteobacteria, Bacteroides, Firmicutes, and Actinobacteria are the phyla that contain more than 99% of the total microbiota [29]. Among these, members of the families: *Enterobacteriaceae*, *Erwiniaceae*, *Yersiniaceae*, *Acetobacteraceae*, *Enterococcacea*, and *Bacillaceae* are the most-frequently described bacteria from the gut of adult *Aedes* spp. (reviewed in [6]).

Numerous efforts have been made to directly modify the mosquito genome in order to limit their ability to transmit pathogens, but there are biological and logistical limitations to overcome and public concerns about safety that must be addressed before these mosquitoes are introduced into the wild population universally [52,57–60]. An alternative approach to manipulating the mosquito genome is the use of microbe symbionts of the mosquito. Paratransgenesis has emerged as a more suitable approach for vector control based on the use of symbiotic bacteria to deliver effector molecules to wild vectors [61]. An understanding of mosquito microbiota is critical for a paratransgenic system approach and includes: 1) the identification of microorganisms that are well established in mosquitoes, 2) are cultivable, 3) are amenable to genetic manipulation, and 4) can be transmitted to the next generation to propagate the desired traits [61–65]. Furthermore, the chosen bacteria should be capable of colonizing a wide variety of mosquito species so that they can be deployed in different species, reducing the need of producing different transgenic mosquitoes for particular environment [52,60,66]. Among bacteria identified in this study, *Pantoea*, *Pseudomonas*, and *Serratia* have been put forward as potential candidates for paratransgenic modifications for vector control strategies [29]. Another candidate for paratransgenesis is *Escherichia coli*, which is not only one of the most abundant organisms in both *Aedes* and *Culex* from Grenada, but also are easy to genetically manipulate, and are culturable. Some studies have already used *E*. *coli* in paratransgenesis systems [60,67]. Unfortunately, it also has been shown that these constructs of *E*. *coli* disappear quickly from the midgut of some mosquitoes which may make them non-suitable for the use in paratransgenic interventions [60]. Some of these symbiotic bacteria may play a critical role in the mosquitoes’ survival [68], and hence, further studies are needed to determine the effect of these bacteria on the two very important populations of *Aedes* and *Culex* mosquitoes.

Other common bacteria found in our study, in the genus *Asaia*, have been found to have permanent association with mosquitoes and are able to quickly colonize tissue in several mosquito species including *A*. *aegypti* and mosquitoes from the *C*. *pipiens* complex [52]. *Asaia* bacteria can be cultured and genetically manipulated. Furthermore, they can be transmitted from males to females during mating [61,68,69].

A potential problem with the use of *Asaia* in mosquito control includes *Asaia* interference with the vertical transmission of *Wolbachia* [27], a bacteria of great importance in mosquito control. According to the literature, co-colonization of the mosquito with *Wolbachia* and *Asaia*, restricts *Asaia* to the gut [6,70]. However, *Asaia* is able to colonize reproductive organs and salivary glands in species uninfected by *Wolbachia* such as *A*. *aegypti* (reviewed in [27]). The exclusion pattern observed between *Wolbachia* and *Asaia* is also found in *A*. *albopictus* and *C*. *quinquefasciatus* naturally infected by both bacteria. [70]. Most studies focus on the microbiota of the gut because of its direct implications with mosquito vector biology [71], so we decided to confirm the location of *Asaia* in *Aedes* and *Culex*. *Asaia* reads were found in both *Aedes* and *Culex* mosquitoes using metagenomics. In agreement with published observations [70,72], *Asaia* from individual mosquitoes was cultured from all *A*. *aegypti* from both the salivary gland and gut, but only from the guts of *C*. *quinquefasciatus*. These observations suggest competition between the *Wolbachia* and *Asaia* for colonization outside the gut but confirm its presence in the guts of both mosquitoes. Therefore, *Asaia* may be a promising candidate for the control of *Aedes* and *Culex*, as reviewed in [6], and confirmed here. Further studies will be required to establish the suitability of *Asaia* as a candidate paratransgenetic organism.

Fungi can also be used in paratransgenic processes [73]. In our study, we found a common fungus belonging to the *Sclerotinaceae* family which was present in both mosquito populations. Many species in this family are plant pathogens which do not affect insects, and therefore make them unsuitable for paratransgenic techniques.

The presence of some bacteria found in this study, like *Serratia* (in both mosquito genera) as well as *Wolbachia* (in *Culex*) are known to provide protective effects from pathogen infections, particularly arboviruses [29,74–78]. Reports on pathogen blocking by *Wolbachia* mainly reflect studies with dengue virus, but there is also evidence of pathogen blocking of other medically important, positive, single-stranded RNA viruses such as Zika, yellow fever, and chikungunya [79–82].

*Wolbachia* is an intracellular endosymbiont naturally present in mosquitoes such as *A*. *albopictus*, *C*. *pipiens*, and *C*. *quinquefaciatus*, but is not thought to be present in *Anopheles* species or *A*. *aegypti* [83,84]. Recently, different *Wolbachia* strains were artificially introduced into *A*. *aegypti* (*w*MelPop‐CLA and *w*Mel from *Drosophila* [85,86], *w*AlbB from *A*. *albopictus* [87], and *w*Mel*w*AlbB [88]) where they formed stable infections. In Grenada, *C*. *quinquefasciatus* was found to have an abundant population of *Wolbachia* among their bacterial microbiota (62.79% REA) while the *A*. *aegypti* population was not found to carry *Wolbachia*. This is consistent with other studies in which *A*. *aegypti* is rarely, if ever, infected with *Wolbachia* naturally, whereas *C*. *quinquefasciatus* is infected throughout its range and in most individual mosquitoes tested [89–91].

*Serratia odorifera*, a species of *Serratia* found in both *Aedes* and *Culex* in this study. *Serratia* secretes *SmEnhancin*, a protein that cleaves off membrane-bound mucins and weakens the peritrophic matrix favoring viral dissemination out of the mosquito midgut, and is thus able to enhance DENV-2 susceptibility in the mosquito [74,92]. *Serratia*-positive mosquitoes were obtained from DENV endemic regions, while *Serratia*-negative mosquitoes were caught in non-DENV-endemic regions supporting the hypothesis that microbiota composition may contribute to the observed differences in vector competence across *A*. *aegypti* populations [93].

A common parasite found in this study was *Albugo*, a common plant-pathogen known as white blister rust. Also, of interest is that *Ascogregarina* was among the parasites found in *Aedes*. *Ascogregarina culicis* is a common gregarine parasite of *A*. *aegypti* [94]. The sporozoites of these parasites invade the midgut epithelial cells and develop intracellularly and extracellularly in the gut to complete their life cycles. The midgut is also the primary site for virus replication in the vector mosquitoes. In previous studies it was found that *Ascogregarina culicis* may have an important role in the maintenance of chikungunya virus during the inter‐epidemic period [94].

The main virus that colonizes *Aedes aegypti* in Grenada is the insect-specific virus belonging to the *Phasivirus* genus, Phasi charoen-like virus [47]. In *Culex*, a very large number of unclassified viruses exist. Of those identified, the *Circoviridae* family was the most abundant, dominated by the animal virus Circovirus, whose natural hosts are pigeons, ducks, and pigs [95–97].

The second most abundant viral family was *Rhabdoviridae*, specifically the Grenada mosquito rhabdovirus 1, a virus first described from a pool of female *Deinocerites* spp. mosquitoes collected in St. John Parish, Grenada, W.I. in March 2015 [42]. To our knowledge this is the first report of this virus in *Culex* spp. Recent publication by Shi et al. (2019) [98] reported a novel virus, the Guadeloupe Culex rhabdovirus in *C*. *quinquefasciatus* that has a 99.36% identity to the previously reported Grenada mosquito rhabdovirus 1 and the one reported in this study.

The third dominant virus was the insect-specific Culex flavivirus (CxFV). CxFV strains have been detected from different *Culex* sp. mosquitoes including *C*. *pipiens*, *C*. *quinquefasciatus*, *C*. *coronator* and others, in many parts of the world [99–107]. Different strains of CxFV have been characterized in the literature [107]. CxFV in natural mosquito populations is maintained by vertical transmission [108].Though one study suggested a positive association between infection with CxFV and WNV [102], other studies found no effect of CxFV on WNV replication, infection, dissemination, or transmission in C. *quinquefasciatus*, as well as no significant correlation between CxFV and WNV infection rates throughout the United States [109,110].

## Conclusions

Our study describes the composition of microbial communities in two common mosquito genera in Grenada, West Indies. that are involved in the transmission of human pathogens. We have identified few common bacteria among the two arboviral-vectors that may be used in mosquito control strategies. The results highlight the importance of these kind of studies in identifying targets for the development of alternative vector control approaches based on microbiota modification. Future studies are warranted to determine the impact of the organisms found here on the growth and vectorial capacity of the mosquitoes, as well as their feasibility for transgenesis or other mosquito control applications.

## Supporting information

S1 Fig Relative estimated abundance of microorganisms as determined by metagenomic analysis: A. *Aedes aegypti* and B. *Culex quinquefasciatus*. (TIF)

S2 Fig Relative estimated abundance of bacteria as determined by metagenomic analysis: A. *Aedes aegypti (*A1. By phyla A2. By class A3 by family, and A4. By genera) and B. *Culex quinquefasciatus* (B1. By phyla B2. By class B3 by family, and B4. By genera).
(TIF)

S3 Fig Relative estimated abundance of fungi as determined by metagenomic analysis: A. *Aedes aegypti (*A1. By family, and A2. By genera) and B. *Culex quinquefasciatus* (B. By family).
(TIF)

S4 Fig Relative estimated abundance of parasites as determined by metagenomic analysis: A. *Aedes aegypti (*A1. By family, and A2. By genera) and B. *Culex quinquefasciatus* (B1. By family, and B2. By genera).
(TIF)

S5 Fig Detection of *Trypanosoma* in mosquito pools by RT-PCR in *Culex quinquefasciatus*. (TIF)

S6 Fig Relative estimated abundance of viruses as determined by metagenomic analysis: A. *Aedes aegypti* (A1. By family, and A2. By genera) and B. *Culex quinquefasciatus* (B1. By family, and B2. By genera).
(TIF)

S7 Fig Detection of flavivirus(es) in mosquito pools by RT-PCR in *Culex quinquefasciatus*. (TIF)

S8 Fig Uncropped S5 Fig (per journal requirements). Top row of gel: First RT-PCR. Bottom row of gel: Second (nested) RT-PCR.
(TIF)

S9 Fig Uncropped S7 Fig (per journal requirements). Top row of gel: *Aedes aegypti* flavivirus RT-PCR. Bottom row of gel: *Culex quinquefasciatus* flavivirus RT-PCR.
(TIF)

S1 Table Primers used in this study. (DOCX)
