# Supplementary figures and images for: Metagenomic analysis of Aedes aegypti and Culex quinquefasciatus mosquitoes from Grenada, West Indies
Source: PLoS One. 2020 Apr 13;15(4):e0231047. doi: 10.1371/journal.pone.0231047 (PMC7153883; doi:10.1371/journal.pone.0231047)

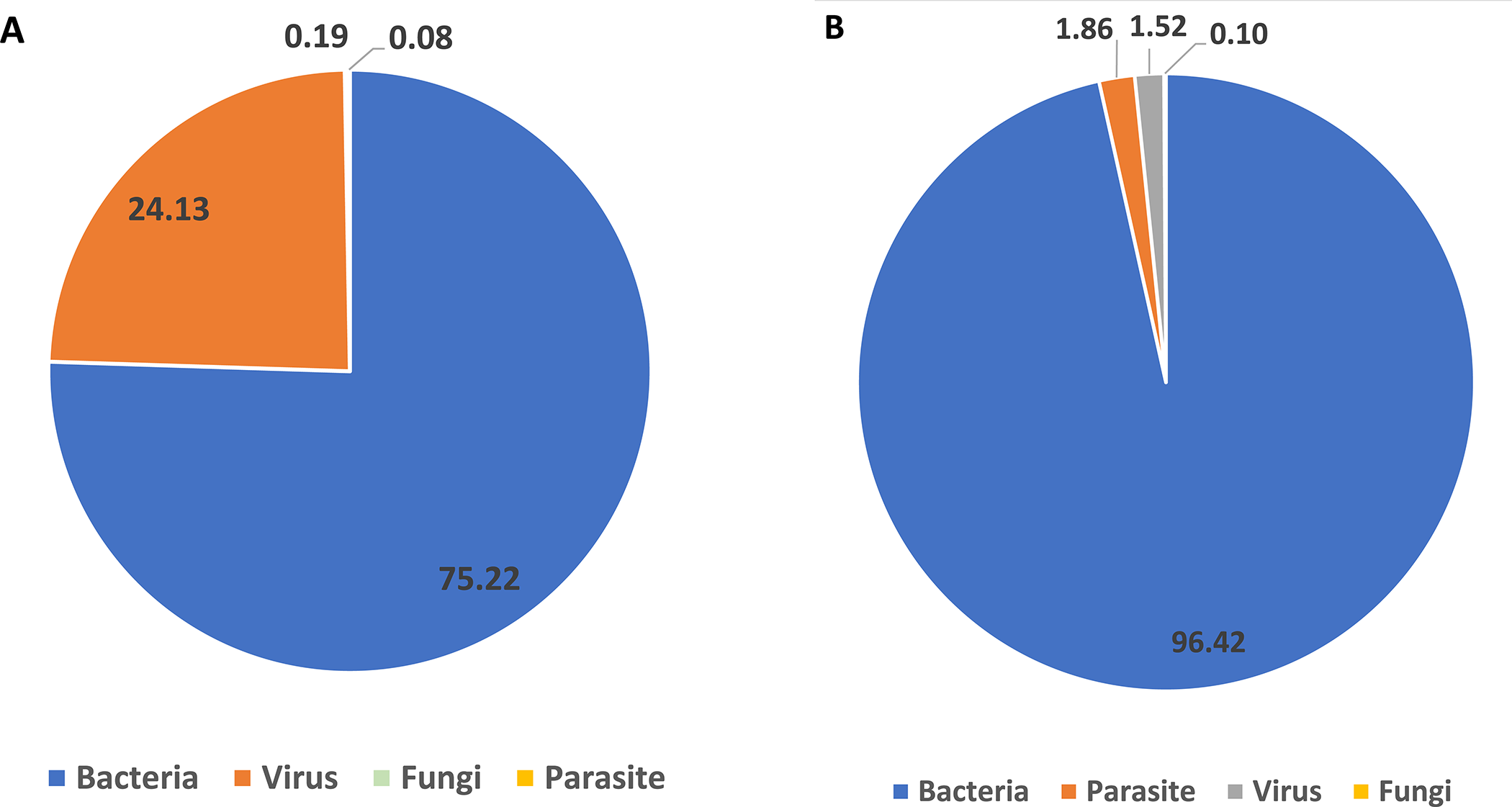

Supplement: S1 Fig — (TIF) [file pone.0231047.s001.tif]

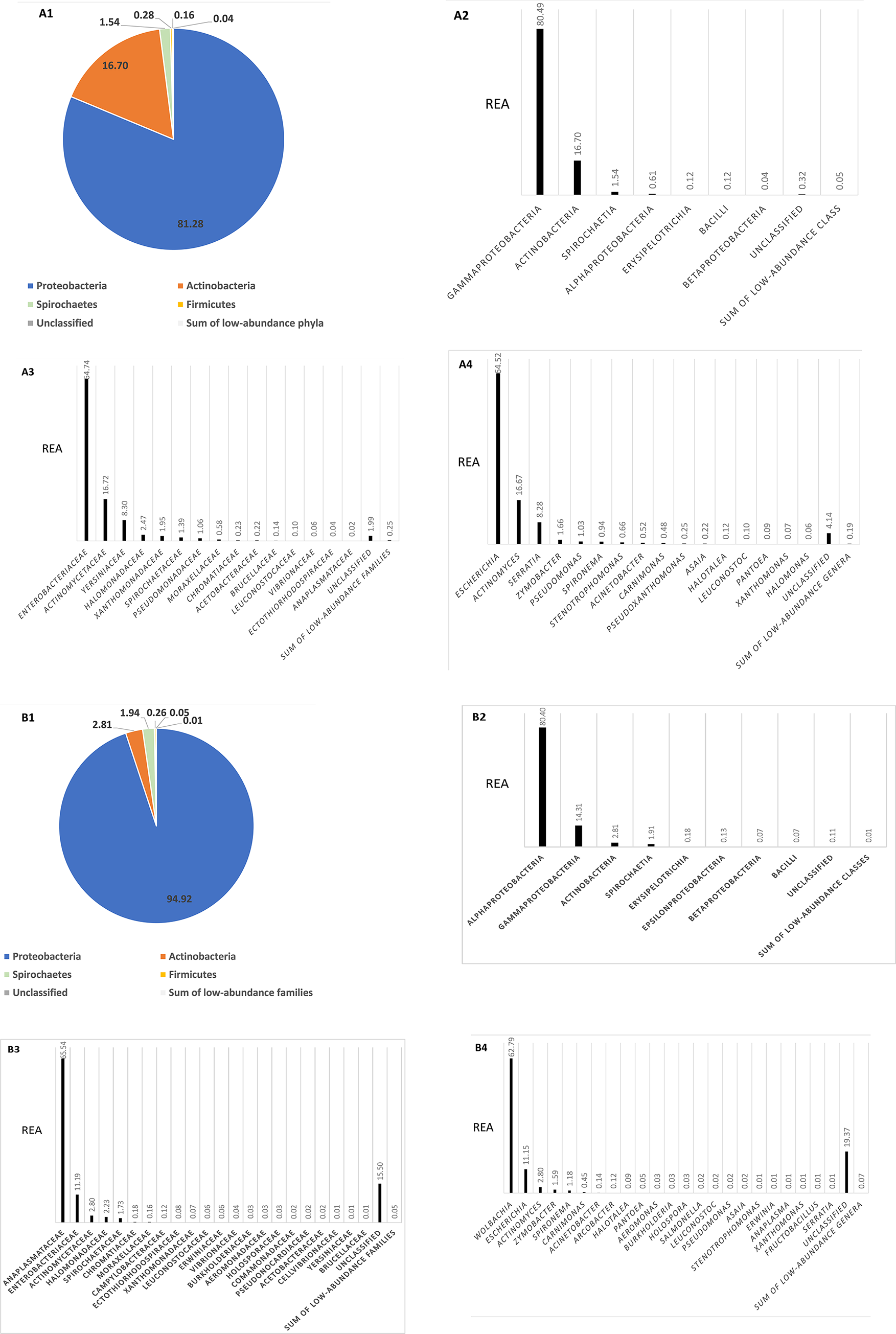

Supplement: S2 Fig — Relative estimated abundance of bacteria as determined by metagenomic analysis: A. Aedes aegypti (A1. By phyla A2. By class A3 by family, and A4. By genera) and B. Culex quinquefasciatus (B1. By phyla B2. By class B3 by family, and B4. By genera). (TIF) [file pone.0231047.s002.tif]

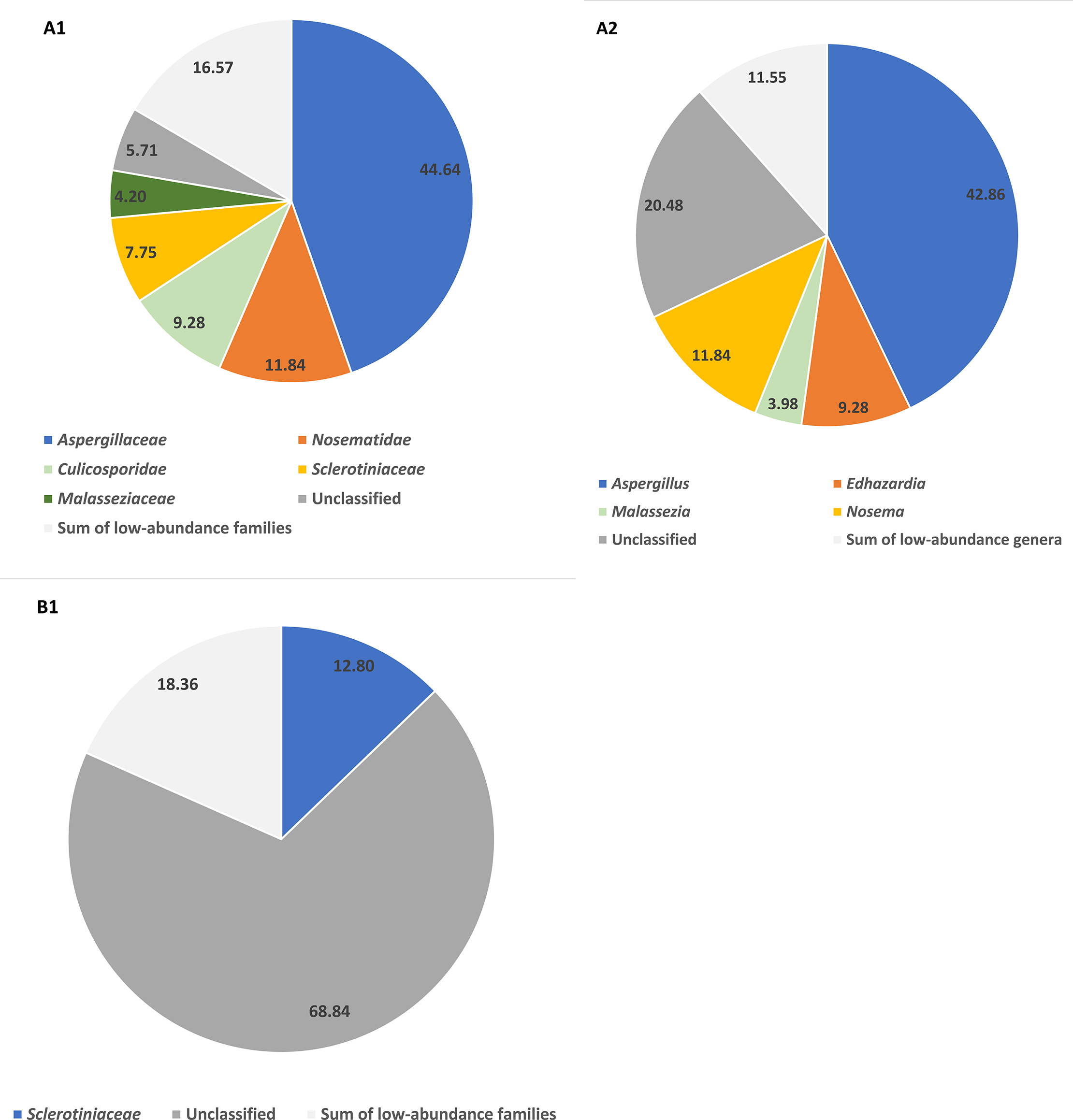

Supplement: S3 Fig — Relative estimated abundance of fungi as determined by metagenomic analysis: A. Aedes aegypti (A1. By family, and A2. By genera) and B. Culex quinquefasciatus (B. By family). (TIF) [file pone.0231047.s003.tif]

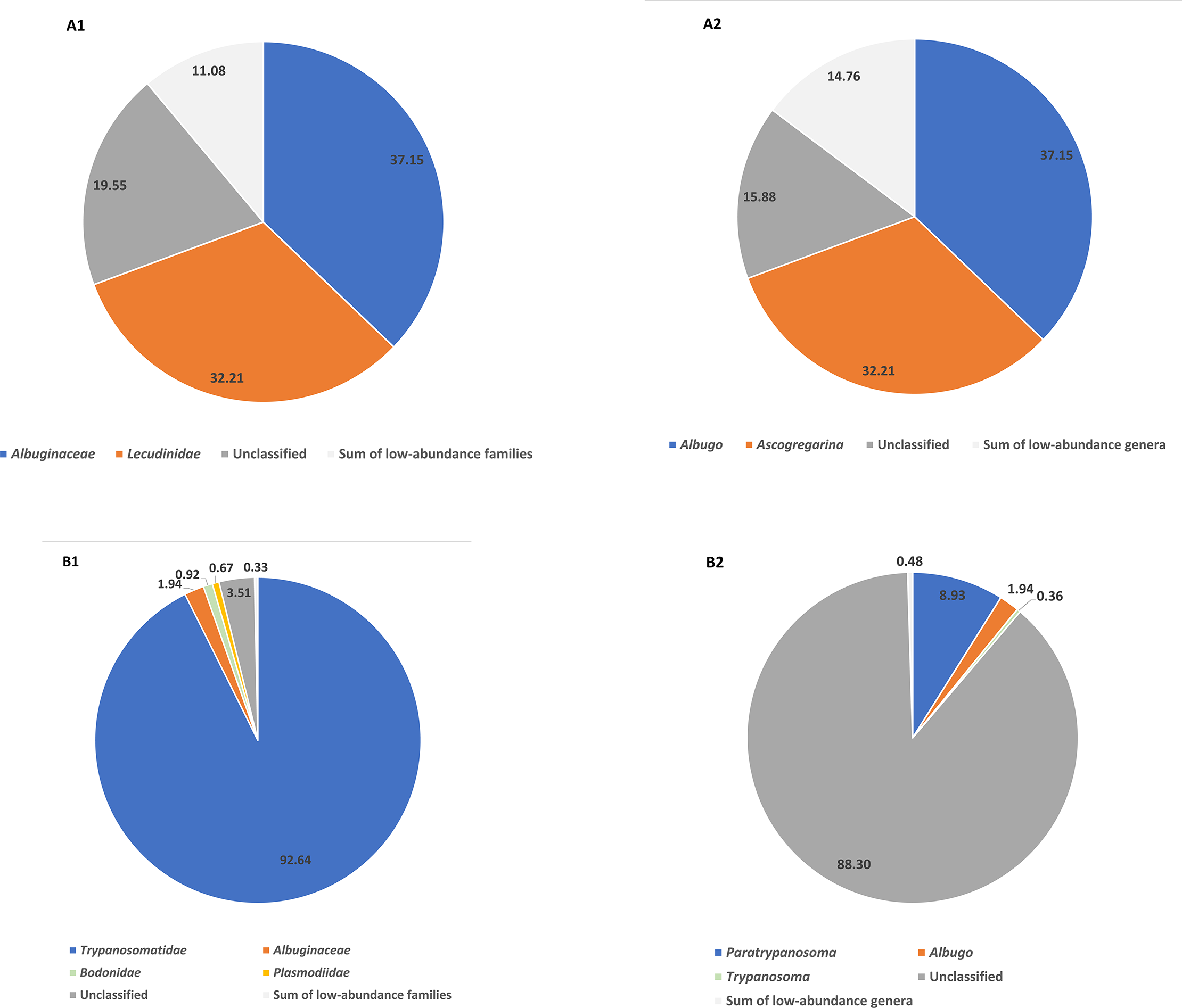

Supplement: S4 Fig — Relative estimated abundance of parasites as determined by metagenomic analysis: A. Aedes aegypti (A1. By family, and A2. By genera) and B. Culex quinquefasciatus (B1. By family, and B2. By genera). (TIF) [file pone.0231047.s004.tif]

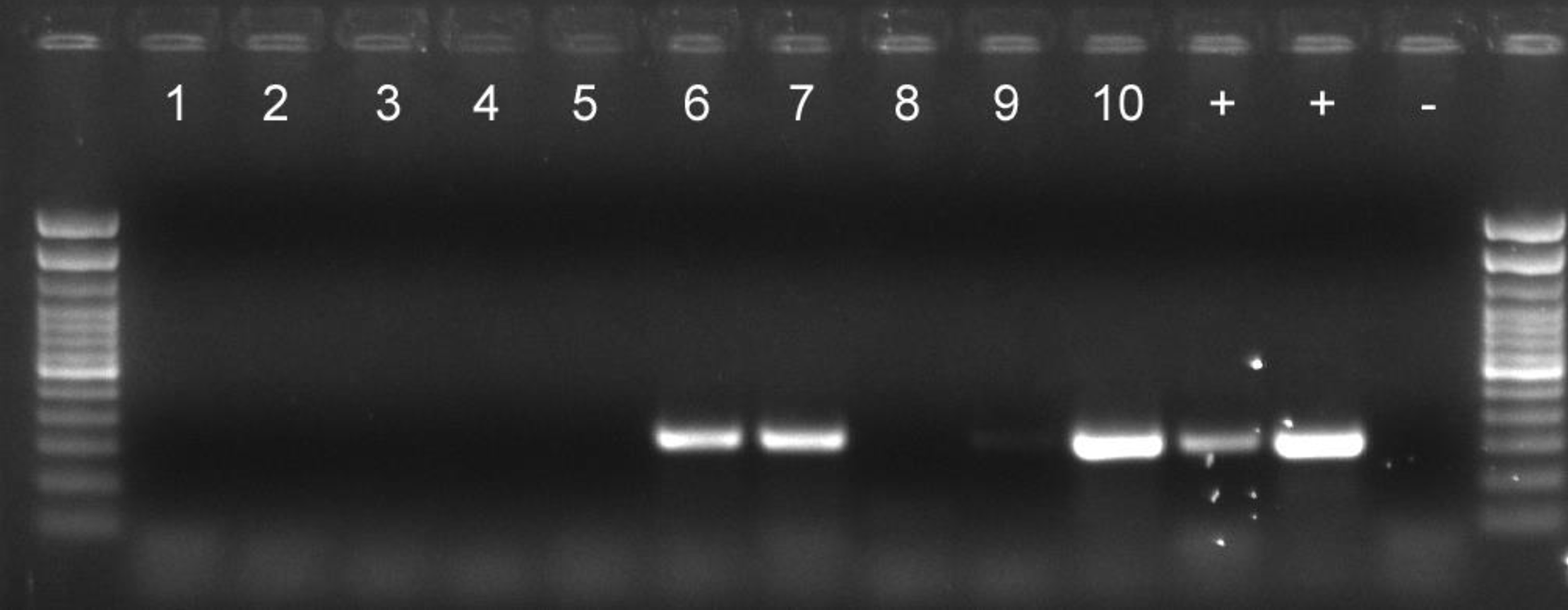

Supplement: S5 Fig — (TIF) [file pone.0231047.s005.tif]

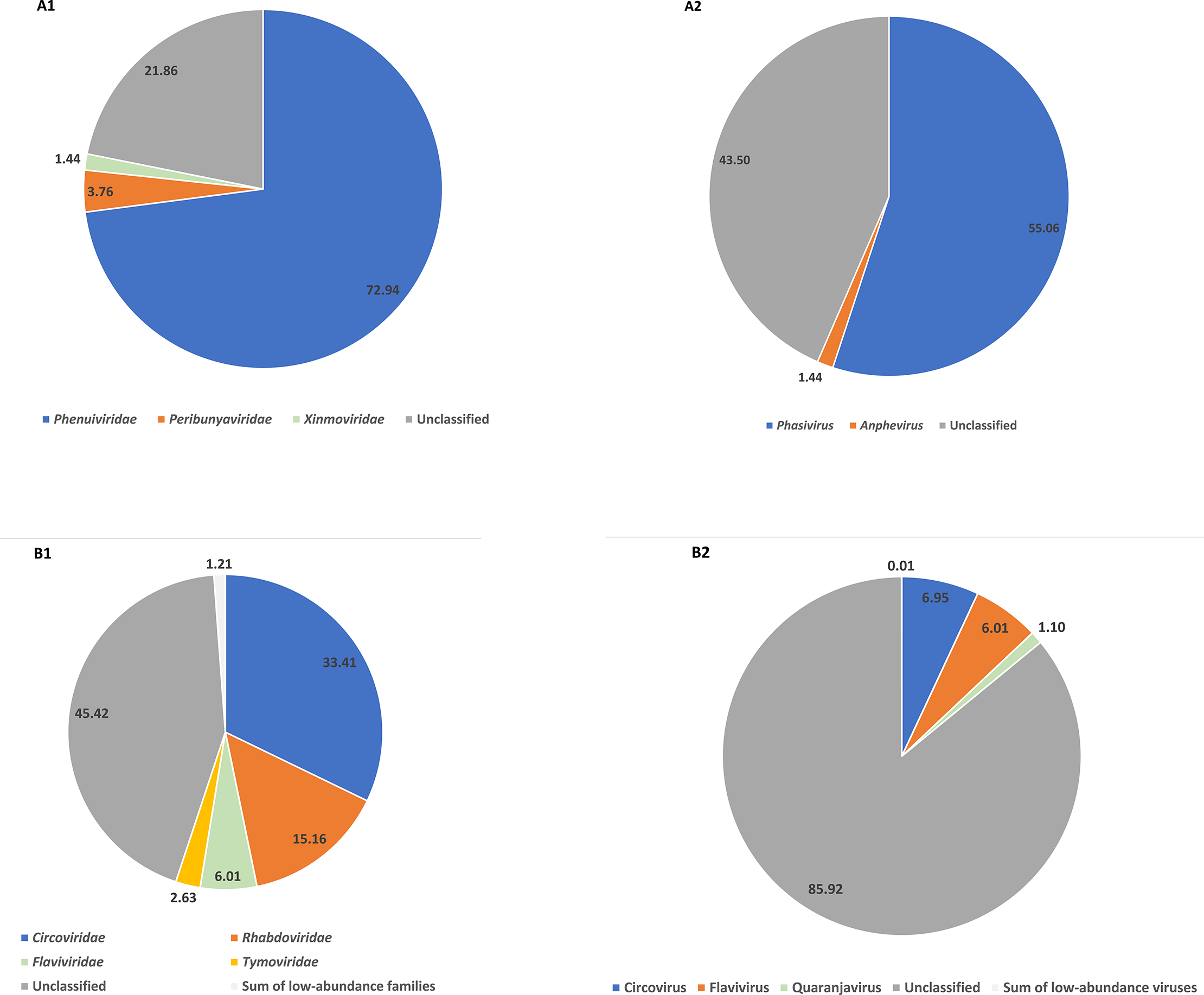

Supplement: S6 Fig — Relative estimated abundance of viruses as determined by metagenomic analysis: A. Aedes aegypti (A1. By family, and A2. By genera) and B. Culex quinquefasciatus (B1. By family, and B2. By genera). (TIF) [file pone.0231047.s006.tif]

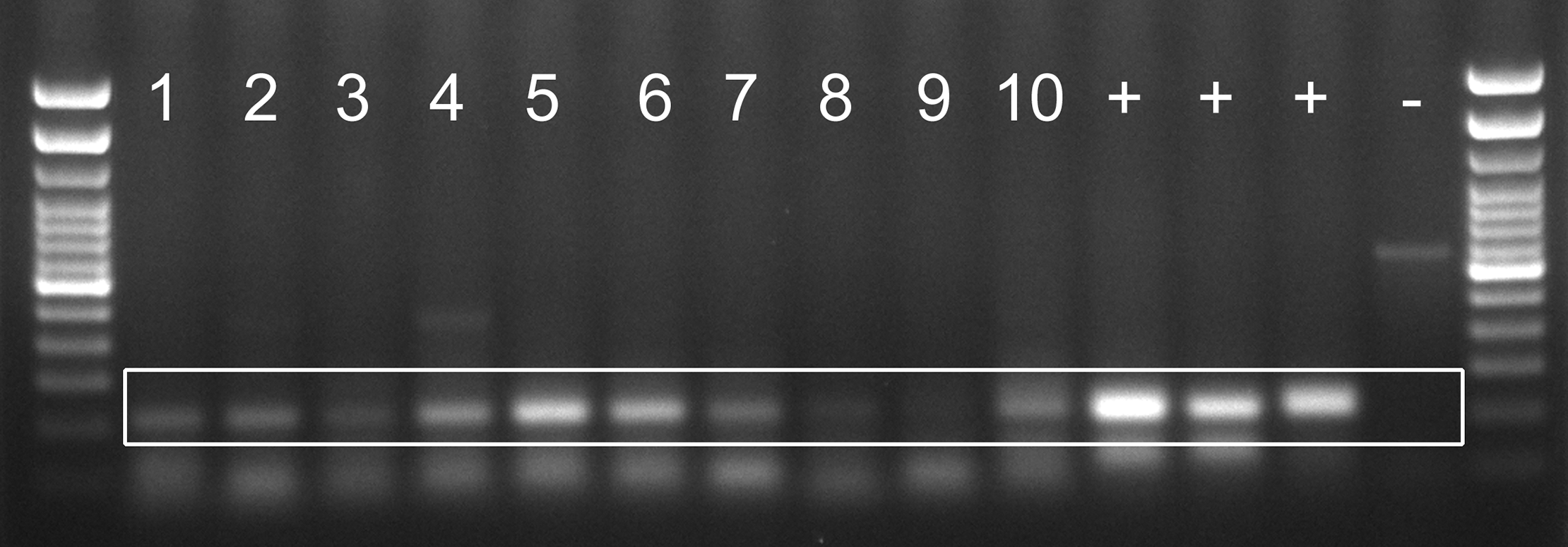

Supplement: S7 Fig — (TIF) [file pone.0231047.s007.tif]

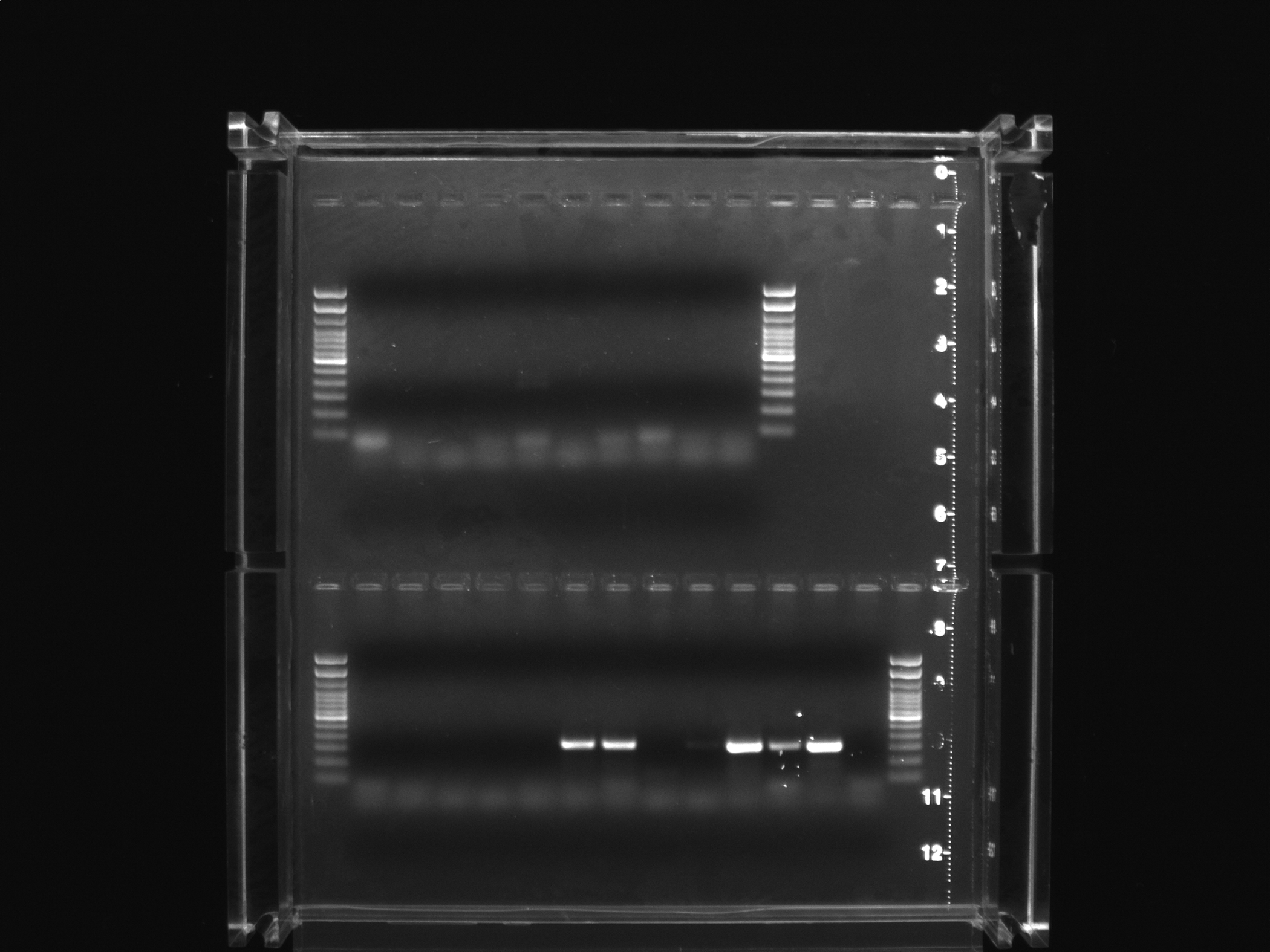

Supplement: S8 Fig — Top row of gel: First RT-PCR. Bottom row of gel: Second (nested) RT-PCR. (TIF) [file pone.0231047.s008.tif]

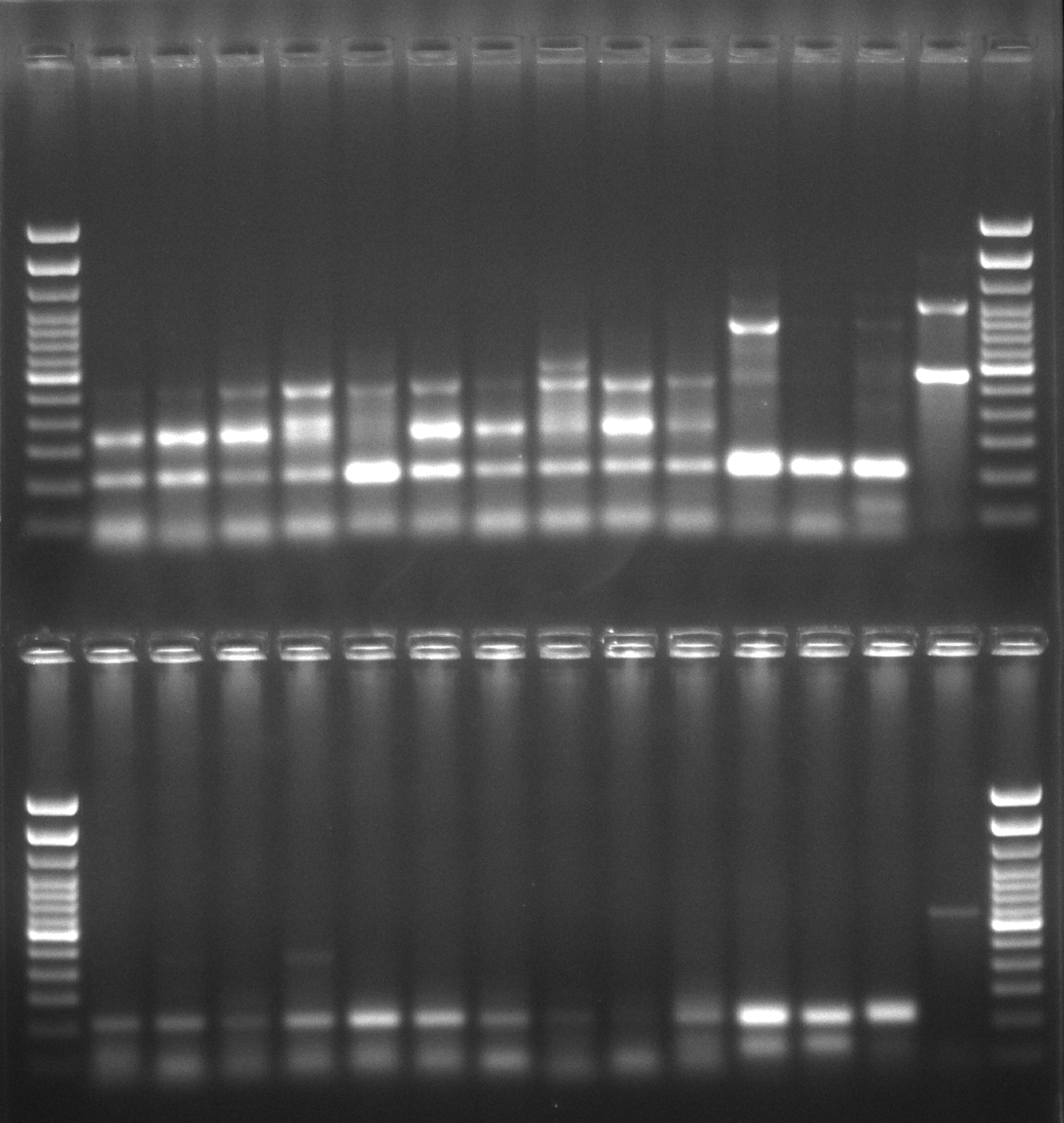

Supplement: S9 Fig — Top row of gel: Aedes aegypti flavivirus RT-PCR. Bottom row of gel: Culex quinquefasciatus flavivirus RT-PCR. (TIF) [file pone.0231047.s009.tif]
