# Supplementary material for: Metagenomic analysis of Aedes aegypti and Culex quinquefasciatus mosquitoes from Grenada, West Indies
Source: PLoS One. 2020 Apr 13;15(4):e0231047. doi: 10.1371/journal.pone.0231047 (PMC7153883; doi:10.1371/journal.pone.0231047)
Supplement: S1 Table — (DOCX) [file pone.0231047.s010.docx]

| Organism | **Primers (5’ to 3’)** | **Target** | **Reference** |
| --- | --- | --- | --- |
| Universal bacteria | 16s2F:  CCTACGGRSGCAGCAG  16s4R: GGACTACCMGGGNTATCTAATCCKG | 16s rRNA | [49] |
| Pan-trypanostomatid | First PCR:  S-762 GACTTTTGCTTCCTCTAWTG  S-763 CATATGCTTGTTTCAAGGAC  Second PCR:  S-755 CTACGAACCCTTTAACAGCA  S-823 CGAAYAACTGCYCTATCAGC | 18s rRNA | [50] |
| Pan-flavivirus | First PCR:  MAMD - AACATGATGGGRAARAGRGARAA  cFD2 - GTGTCCCAGCCGGCGGTGTCATCAGC  Second PCR:  FS 778 - AARGGHAGYMCDGCHATHTGGT  cFD2 - GTGTCCCAGCCGGCGGTGTCATCAGC | NS5 | [51] |
